# Systematic experimental investigation of the obstacle effect during non-competitive and extremely competitive evacuations

**DOI:** 10.1038/s41598-020-72733-w

**Published:** 2020-09-29

**Authors:** Claudio Feliciani, Iker Zuriguel, Angel Garcimartín, Diego Maza, Katsuhiro Nishinari

**Affiliations:** 1 Research Center for Advanced Science and Technology, The University of Tokyo, 4-6-1 Komaba, Meguro-ku, Tokyo, 153-8904 Japan; 2 Departamento de Física y Matemática Aplicada, Facultad de Ciencias, Universidad de Navarra, Pamplona, Spain; 3 Department of Aeronautics and Astronautics, Graduate School of Engineering, The University of Tokyo, 7-3-1 Hongo, Bunkyo-ku, Tokyo, 113-8656 Japan

**Keywords:** Civil engineering, Applied mathematics, Nonlinear phenomena, Phase transitions and critical phenomena, Statistical physics

## Abstract

Although some experimental evidence showed that an obstacle placed in front of a door allows making people’s evacuations faster, the efficacy of such a solution has been debated for over 15 years. Researchers are split between those who found the obstacle beneficial and those who could not find a significant difference without it. One of the reasons for the several conclusions lies in the variety of the experiments performed so far, both in terms of competitiveness among participants, geometrical configuration and number of participants. In this work, two unique datasets relative to evacuations with/without obstacle and comprising low and high competitiveness are analyzed using state-of-the-art definitions for crowd dynamics. In particular, the so-called congestion level is employed to measure the smoothness of collective motion. Results for extreme conditions show that, on the overall, the obstacle does not reduce density and congestion level and it could rather slightly increase it. From this perspective, the obstacle was found simply shifting the dangerous spots from the area in front of the exit to the regions between the obstacle and the wall. On the other side, it was however confirmed, that the obstacle can stabilize longitudinal crowd waves, thus reducing the risk of trampling, which could be as important (in terms of safety) as improving the evacuation time. However, under urgent, competitive, but non-extreme conditions, the obstacle generally had a positive effect, helping channeling the flow of pedestrians through the exit while facilitating their interactions.

## Introduction

The 20th century has seen rapid demographic changes both in terms of human population and its distribution. As the overall world population increased, the more and more people moved from rural to urban areas and cities have been challenged with the task of managing a large population in a limited space. The trend has not changed with the turn of the century with even more people moving to live and work in densely populated cities. In many cities urban transportation network is often overloaded or running close to capacity with dense crowds typically forming during rush hour or in case of service interruptions. The lack of available land surface makes it difficult to broaden available structures, especially in locations close to central districts, which also tend to be the most crowded. An optimal crowd management is therefore becoming of primary importance to ensure safety in pedestrian facilities with simple, effective solutions increasingly sought^1^.

In this context, it should be remarked that although crowd management only recently became relevant to the daily life of many people, occasional accidents have occurred for centuries (one of the earliest being accurately reported is the Shiloh Baptist Church stampede of 1902 where 115 people lost their lives^2,3^). Every year stampedes are reported in different parts of the world taking the lives of thousands of people^4–6^. Most stampedes occur during special (often religious) events, when a large number of people gather in a small place for a short time^7^. Causes of those accidents are often related with organizational/planning issues^8–10^, typically due to a lack of preparation or missing coordination between the different stakeholders^11,12^, but a wrong estimation over the number of participants (sometimes caused by a lack of control over the number of tickets issued^13,14^) is also a common cause.

While organizational failures are difficult to prevent and are unfortunately often discovered after tragedies already occurred, constructional elements could be used to ensure a safe motion of crowds also under the worst situation. Stadia, train stations, exhibition halls and large venues where large crowds constantly gather are specifically designed on that purpose and internal structures are optimized for pedestrian motion^15,16^. However, as already said, modifications are difficult in large structures^17^ or are possible only during large renewal works (when design is largely revised) and temporary events usually miss the resources to perform an adequate study on pedestrian flows.

Even though every pedestrian facility is different and peculiar in its structure, there are some common elements which are found everywhere. Corridors, corners or crossings are some examples for these fundamental elements. However, one of the most critical elements of pedestrian traffic is represented by bottlenecks^18^. The number of bottlenecks should be generally minimized when designing pedestrian facilities, but it is not always possible to avoid them at all. Bottlenecks are particularly dangerous because pedestrian flow is reduced and life-threatening crowd densities may form in front of them^19–21^.

A simple, universal and counter-intuitive method which has been proposed to reduce the risk of pedestrian accidents at bottlenecks is to place an obstacle right in front of it^22^. It is argued that the obstacle (circular ones seemingly the most effective) can help reducing the forces caused by people pushing toward the exit (creating the so-called arch effect). Although the advantages of such a solution should be clear especially when considering the discussion above (it is easy to implement also in already completed or temporary structures), its efficiency has been debated for years among researchers.

Without going into details (an exhaustive review of studies considering the obstacle effect is given by Shiwakoti et al.^23^) researchers are typically divided into those who found the presence of the obstacle beneficial and those who found it worsening pedestrian safety (or ineffective in terms of safety). Even among those who found beneficial effects experimental/geometrical conditions are often different. Some researchers performed their studies under non competitive conditions and low density crowds while others tried to reproduce conditions close to emergencies. Distance (close or far from the exit), position (centered or shifted respect to the exit), size, shape and number of obstacles were also found relevant for overall efficacy.

While all experimental studies performed so far definitely helped researchers in the understanding on why obstacles can/cannot be beneficial, two important aspects have been little considered in the past:

- **Extremely competitive pushy behavior.** Many experimental works considered competitive behaviors by having participants rushing toward the exit, but, due to safety and ethical concerns, specific instructions were given to avoid participants pushing and/or the number was limited to avoid the build up of high pressures close to the exit. Also, experiments targeting extreme conditions, usually focused only on those conditions, without comparing the role of obstacle under different level of competitiveness. In order to fill this gap Garcimartín and Zuriguel^24–27^ performed novel experiments in which students and soldiers were employed to create conditions possibly close to “real” emergencies. However, both datasets were never compared in a systematic way, thus limiting the extent of the conclusions which could be learned from their experiments.
- **Congestion level and coordination within the crowd.** The vast majority of the studies performed so far focused on the concept of “density”. Several definitions of density have been used but all focus on determining the concentration of pedestrians in a particular region^28^. Density has therefore a static nature and, despite being obviously important and useful, cannot shed light on some relevant properties of pedestrian motion, such as “smoothness”, for instance. To overcome this problem, the so-called “congestion level” has been defined to measure the capability of crowds to move in an organized way^29^, potentially limiting the risk of accidents. Being a new definition, implementations have been only limited to few small-scale evacuations without obstacles^30^.

In this work, we wish to overcome the issues discussed above by considering a series of experiments ranging from “normal” to extreme conditions and employ the latest methods presented for crowd dynamics in analyzing the results.

This work is organized as follows. In the next section experiments and analyzed datasets are presented; then, empirical methods are introduced and the results are presented. Finally, some conclusions are discussed. Minor details on data analysis and/or less relevant results are given in the supplementary material.

## Experimental setup and procedures

**Figure 1:**
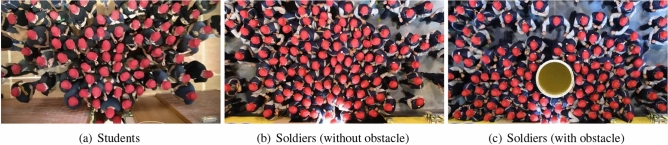
Representative frames for the experiments considered in this work. All frames were taken shortly after the start of the evacuation.

This work is based on two separate experimental trials which have been already described in other publications. For the sake of brevity, we will therefore present here only those information which are fundamental for the understanding of this work and provide references to the original works for readers interested in more technical details. Figure 1 presents three images representative of the experiments that will be described hereafter.

### Experiments with students

In the first set of experiments (details are provided in^24^) evacuation drills were conducted using 95 volunteer students as participants. A mockup room with a 69 cm exit was prepared and participants were instructed to wait in an area of $6 \, \hbox {m} \times 4 \,\hbox {m}$ in size (with the exit at the center of the longest side) before the start of each trial (initial density was consequently roughly 4 persons ${\hbox {m}}^{-2}$). After a signal was given, participants were asked to leave the room passing through the single exit. Three different instructions were given in terms of competitiveness:

- **Low competitiveness:** participants were asked to avoid intentional physical contact.
- **Medium competitiveness:** soft physical contact was allowed, but pushing was forbidden.
- **High competitiveness:** moderate pushing was allowed (excessive pushing was still forbidden for safety reasons).

In total 10, 9 and 13 trials were performed for the low, medium and high competitiveness case respectively. All experiments have been supervised by safety officials who could interrupt the execution should any dangerous situation occur. In addition, particular care was taken to have participants acting individually and not behaving in groups to reduce the number of variables potentially influencing crowd behavior.

A camera was placed in azimuthal position above the exit and trajectories of participants were extracted from videos using the red caps wore by participants as marker. Datasets including videos and extracted trajectories are available at^31^.

### Experiments with soldiers

Although the experiments with students already considered highly competitive conditions, pushy behavior was restricted for safety and ethical concerns (students could easily get hurt). To allow recreating conditions similar to a real emergency evacuation while ensuring the safety of participants, a second set of experiments was performed by involving professionally trained soldiers of the Spanish army. In this second experimental campaign (details on these experiments are given in^26^) 181 soldiers were recruited as participants. However, since soldier had the possibility to opt out at any time, the number of participants for each trial varied between 165 and 181.

Similarly to the experiments with students, also soldiers had to collect in a $10 \, \hbox {m} \times 5 \, \hbox {m}$ area in front of a 75 cm door and evacuate through it once the start was given (initial density was thus comparable to the students’ case). However, in contrast to the case with students, soldiers were allowed to push, thus resulting in what is possibly one of the controlled evacuation experiment closest to real emergency conditions. Still, to ensure safety and avoid that injuries would occur (which, for instance, are not rare in airplane evacuation tests^32,33^), officers were supervising the experiments and could stop them at any moment should a dangerous situation occur. This occurred a couple of times indeed and those executions had to be stopped as some participants got their arm trapped at the doorjamb or were about to fall down. However, all experiments were successfully concluded without injuries being reported.

Level of competitiveness was also varied in this experimental campaign, but only in a two step fashion by giving/refusing to participants the possibility to intentionally push. From this aspect, the no pushing/pushing condition was said to be comparable to the low/high competitiveness condition for students, respectively.

In addition, a cylindrical obstacle of 100 cm in diameter was used in some of the trials to test its efficacy during evacuations. The resin-made obstacle had been filled with water and weighted around one ton, thus making it very difficult to displace it even under extreme crowd pressures. Distance from (the external surface of) the obstacle and the door was set at 50, 60 and 70 cm in different trials.

To summarize, even though competitiveness was the only variable in the experiments with students, in the case of soldiers three variables were considered, namely: degree of competitiveness, presence of the obstacle and its distance from the door. Aiming a good statistical reliability, each experiment was repeated several times; the number of repetitions being higher for competitive conditions where the variability of the results was proved to be more important (see Table 1).

**Table 1:** Number of repetitions for each configuration in the experiments with soldiers.

Competitiveness	Presence of obstacle	Obstacle distance	Number of repetitions
Low	No	N/A	3
Low	Yes	60 cm	3
High	No	N/A	7
High	Yes	50 cm	6
High	Yes	60 cm	6
High	Yes	70 cm	6

Analogously to the experiments with students, also in the case of soldiers, video recordings were taken by placing a camera right above the exit and trajectories were later extracted by video processing. Trajectories and videos relative to the experiments with soldiers are provided in^34^. Finally, in addition to the video camera providing tracking information, in the experiments with soldiers a pressure sensor was mounted on the doorjamb (technical details are provided in^27^), thus allowing to measure the pressure exerted on the wall from the evacuating crowd.

## Analytical methods

Despite some differences in experimental procedure, datasets for student and soldier experiments both consist in participants’ trajectories (the only minor difference between both datasets consist in the frame rate of the videos, being 50 FPS and 25 FPS for the student and soldier experiments respectively) and have been analyzed using the procedures described in this section. Some of the methods presented here are original for this work, but others are based on previously implemented analysis. Regardless of this we will try to provide a summary overview for all methods to allow readers getting a sufficient understanding while reading this work.

This section has been mainly divided into two parts to distinguish analytical methods which can be generally used to analyze any sort of crowd in any geometrical configuration (universal measures) and those methods which are specifically (or particularly) designed to study the bottleneck condition (specific measures).

### Universal crowd measures

#### Density by Voronoi diagram

Density is one of the most important measures for crowd dynamics and closely related to pedestrian safety. Several methods have been proposed to measure density, although the Voronoi diagram method has been found being the most accurate, effectively reproducing the “personal space” of pedestrians also in low-density conditions^28,35^. Using the Voronoi method it is possible to obtain the “personal space” for each participant by computing the surface of her/his corresponding Voronoi cell. Density is consequently defined by taking the inverse of the Voronoi cell’s surface.

**Figure 2:**
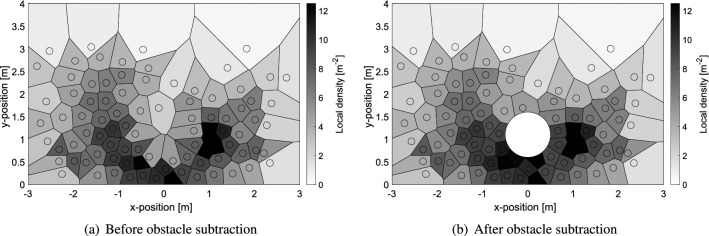
Subtraction of the obstacle’s surface from Voronoi cells relative to its location. The gray scale is used to indicate the local density for each participant. Density of participants close to the obstacle is clearly affected by the modification of their cells.

Using this method, each pedestrian’s position can be assigned with a local density in each frame. However, when the obstacle is used, it is necessary to remove its area from the Voronoi cells overlapping it. Therefore, when analyzing the experiments with the obstacle, its portion of the area has been removed from the affected Voronoi cells^36^. The difference resulting in the subtraction of the obstacle is shown in Fig. 2 for a typical frame of the experiment with soldiers.

As a closing remark on density, it should be noted that the Voronoi method provides accurate results only when the position of the evacuees is known past the exit. Since this was not the case in the experiments considered here, the density close to the exit cannot be considered accurate. However, considering the overall gain in accuracy generally provided by the use of the Voronoi method, the imperfections close to the exit can be considered an acceptable drawback.

#### Congestion level and crowd danger

**Figure 3:**
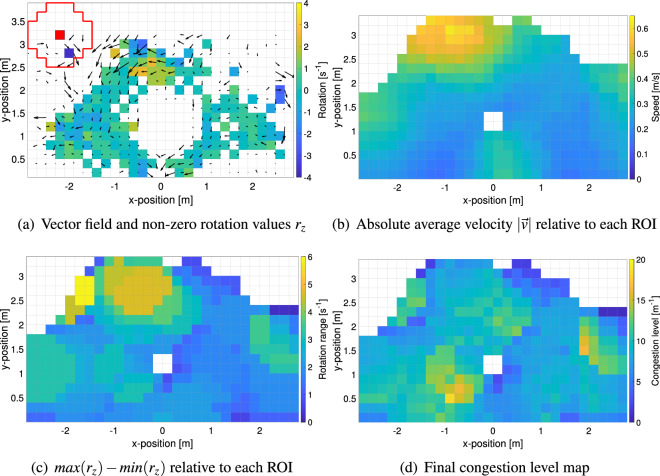
Illustration of the steps followed for computing the congestion level. As it can be seen, higher levels of congestion are reached when a moderate rotational movement of the crowd is seen in correspondence to a small velocity, thus implying that people may be simply moved around by the force of the crowd without being able to impose their own moving direction.

The so-called congestion level has been recently proposed as a new measure with the aim to universally quantify the “motion smoothness” in pedestrian crowds^29,30^. Several definitions have been already proposed in the past on the same scope, but either they focused on specific cases (like the Level of Service^37^ or the order parameter^38,39^) or have not been tested on multiple scenarios (like the “crowd pressure”^40^). The congestion level is computed in several steps summarized as follows (more details, including parameters’ selection, are provided in^29^):

1. The area analyzed is discretized into a mesh with cells having a side length of 0.2 m.
2. Using a sampling time of 2.5 s trajectories are analyzed to determine the average velocity vector inside each cell. If multiple trajectories occur to pass within a cell, the average velocity of all trajectories is considered. A resulting vector field obtained from a selected experimental frame is shown in Fig. 3a.
3. The rotation (curl) is computed over the whole vector field (a central differences scheme is employed) excluding areas containing empty cells (where calculation would not be possible). The result is a matrix containing the only non-zero values of the rotation $r_z$ (perpendicular to the surface). Values for the rotation are indicated in Fig. 3a using a color scale.
4. Using a circular ROI (Region of Interest) as shown in red in Fig. 3a the local congestion level $Cl = \frac{max(r_z) - min(r_z)}{\left| \vec {v} \right| }$ is computed within the ROI, with $\left| \vec {v} \right|$ being the average absolute velocity inside its boundaries. The resulting local *Cl* value is then assigned to the central cell (calculation is skipped if there are less then two non-zero values within the ROI). The ROI is moved cell by cell until the whole surface is covered (in external regions the ROI in considered in half for edges and one quarter for corners). The result of this operation is shown in Fig. 3d, with Fig. 3b,c showing the denominator and numerator component of the congestion level equation respectively.

The congestion level quantifies the capacity of crowds to move in an organized and synchronized way while keeping a considerable moving speed. For instance, a group of people moving in exactly the same direction in a perfectly aligned way, would result in a congestion level equaling zero. On the other side, the highest values of congestion level are reached when crowds tend to show a rotational movement without actually moving to any particular direction (what has also been labeled as “crowd turbulence”^40^).

Being based on a velocity vector field, the congestion level does not consider the concentration of people. For instance, if people move perfectly aligned, the congestion level will be zero regardless on the number of people. However, also density plays an important role. Taking the previous example of people moving in group in the same direction, if, for instance, one person will loose synchronization and would start colliding with others (in that case congestion level starts increasing), the density of people makes a difference in the gravity of the situation. The crowd danger *Cd* is therefore defined as the product of the congestion level with density: $Cd = Cl \cdot \rho$. Calculation of the crowd danger is performed for each cell by multiplying the Voronoi density for a pedestrian located within a given cell with its corresponding congestion level.

As a closing remark on both quantities discussed here, a few words are necessary in regard to their naming and their units. Names for congestion level and crowd danger have been chosen considering their potential to quantify congestion (or collective disorganization) and the risk of crowd accidents. Although the name selection is arguable, there is no doubt that units of both quantities do not reflect their names. We would like therefore to clarify that names have been assigned to both quantities to simplify discussion and presentation of the results, but care should be taken when generalizing the connection between names and units (for instance, “danger” typically has no units and if one had to be assigned, ${\hbox {m}}^{-3}$ would probably not be a good choice). In addition, to partially explain the motivation behind the names used in this work, we would like to mention that we decided to stick to definitions used in the previous works where both quantities were first introduced^29,30^.

#### Floor distribution maps of crowd quantities

Calculations presented so far all considered a single experimental frame or a small portion of each trial. However, all trials lasted for roughly one minute and they have been repeated several times. To simplify the comparison of the different conditions (level of competitiveness, presence of obstacle, etc.) and to reduce the results to the most salient features, the following procedures have been used to obtain a map relative to the spatial distribution of each crowd quantity.

1. Each trial was examined by considering the time between the first and last participants leaving the door. As a short remark, it should be noted that, in some studies^41,42^, a few participants have been removed at the beginning/end in the analysis to consider only steady-state conditions or to avoid the effect caused by uncompliant participants reluctant to move. This is a necessary precautionary measure when overall evacuation time is the main result^42^, if number of participants is particularly low^41^ or if participants are under-motivated and not willing to rush toward the exit. Since none of these conditions apply to the experiments examined here, all participants have been used in the analyses.
2. The experimental surface (or better said the area covered by the camera) has been divided into a mesh having 0.2 m in size where density, congestion level and crowd danger have been computed. Density maps were computed at every frame (thus every 0.02 s for students and every 0.04 s for soldiers) and congestion level and crowd danger were computed every 0.2 s. Later, the average map representing the distribution of the three quantities in each trial was computed taking the average over all frames considered.
3. Finally, all trials relative to the same experimental condition were averaged to get a single map representing the distribution of density, congestion level and crowd danger during the evacuation. To remove the noise (sometimes occurring in external regions with few participants) a local linear regression was performed on the surface obtained.

### Bottleneck specific measures

Although the three quantities presented above already allow performing a systematic analysis of the experiments, there are some aspects which would need a more detailed investigation and, in that regard, more specific measures will have to be developed. More concretely, it can be said that the congestion level provides an estimate on how much people are collectively organized, but its derivation relies on a short time scale. By observing the videos of the experiments, it can be noted that at high levels of competitiveness, shock waves are observed in which people collectively oscillate in a direction parallel to the wall with low frequencies. The congestion level would result in low values if people move in group in the same direction, thus implying an organized condition. However, in evacuations, people in the outer parts of the crowd may trample because of the oscillations. To investigate this kind of “pressure waves” it is necessary using a different approach which considers oscillations on larger time scales.

#### Distance ratio

**Figure 4:**
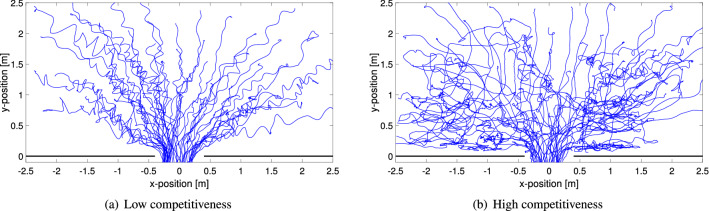
Trajectories for two selected experiments with students at different levels of competitiveness. Only 50 random trajectories are selected to allow a better evaluation of qualitative features. Short range fluctuations are created by the swaying of the body.

One of the simplest methods to estimate the extent of crowd oscillations is to compute the ratio between the minimum distance to the exit (assumed at the coordinate (0, 0)) and the effectively walked distance (what is also called tortuosity). It can be easily assumed that when people are moved around from the collective force of the crowd they will not be able to reach the exit with the shortest path and will have to travel a longer distance. A quick observation on the trajectories for different levels of competitiveness as provided in Fig. 4 clearly shows that in competitive conditions longer and less straight trajectories are common.

**Figure 5:**
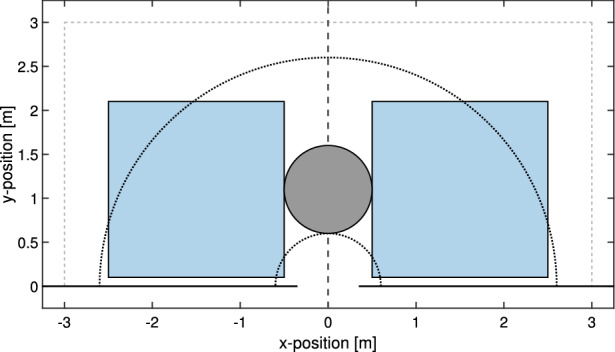
Division of the room in right and left side and regions used to investigate crowd stability and related quantities. In this example the obstacle is at 60 cm from the exit. The semicircular area is used to define participants whom distance ratio and side change ratio is computed (respect to their initial positions). The squared areas on both sides of the obstacle are used to compute velocity and alignment parameters fluctuation.

However, this simple indicator has two disadvantages. One being the fact that people could decide on their own willingness to take longer paths (especially in the outer part of the crowd), thus limiting the capability to measure instabilities. This problem can be solved by considering only an “internal” portion of the crowd, where people are constrained in their motion by others. As a consequence, only a semicircular area between the (front side of the) obstacle and 2 m away from it has been considered (see Fig. 5). Thus, only pedestrians present in that area at the beginning of each experiment have been used to compute the distance ratio. However, as a second disadvantage, it may not be possible to perform a comparison with the obstacle case, as shortest paths are longer by definition when the obstacle is present. Since this issue cannot be solved easily, an additional measure was defined.

#### Side change ratio

Another method to estimate stability, or, in this case, the “orderliness” of the crowd could consist in computing the proportion of people starting on one side but leaving from the opposite side. On this scope, the experimental area is divided into two sides as shown in Fig. 5 and it is determined on which side each pedestrian was at the start of the experiment. The final side is determined taking the position 0.5 s before transiting the exit and the proportion of pedestrians “switching” side is computed.

Being symmetrical in regard to the obstacle, this method allows a comparison with the case without the obstacle. Again, to avoid problems related with people moving in the empty external area of the crowd, only the region contained with the semicircle given in Fig. 5 was considered, or better said, only the people starting in that area have been used to compute the side change ratio.

The two measures introduced above already allow to perform an initial investigation on crowd long-term instabilities, but to better study the phenomenon two additional measures are introduced. This time, two regions lying to the left and the right side of the obstacle as indicated in Fig. 5 are considered. The size of the two areas is identically of $2 \, \hbox {m} \times 2 \, \hbox {m}$. To ensure that both areas are filled with people and thus consider the internal dynamics of the crowd, only the initial 3/4 of the evacuation time is considered from now on (start and end of the evacuation are defined as discussed above using the first and the last participant leaving the door, respectively).

#### Velocity fluctuation

In each of the squared blue areas of Fig. 5 the average *x*- and *y*-component of the velocity has been computed considering the participants within them. To avoid the effect of body swaying (clearly visible in Fig. 4) a time interval of 2.5 s (the same used in the congestion level) was used to compute the average velocity of each participant and later get the average of each area.

**Figure 6:**
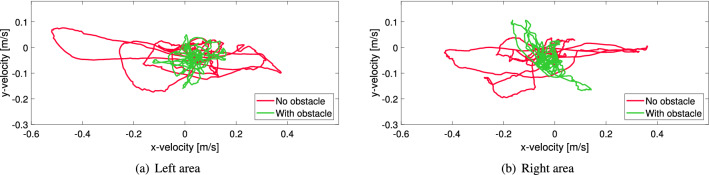
Relation between the average group *x*- and *y*-velocity in the regions of Fig. 5 for two selected experiments with the army. Both experiments had a high level of competitiveness and the distance of the obstacle (when employed) was 60 cm.

Figure 6 shows the relationship between the *x*- and *y*-velocity during the evacuation process in both areas for two representative trials with and without the obstacle (thus experiments with soldiers are used). As expected, *y*-velocities are mostly negative as people move to the exit, but a large range is seen for velocities in the *x*-direction, especially in the case without obstacle. To quantify the extent of these velocity oscillations, we measured the length of each curve and divided it by the considered duration of the evacuation. What we defined as the “velocity fluctuation” can be expressed as:

1 $$\begin{aligned} \phi _v = \frac{1}{I \cdot \Delta t} \displaystyle \sum _{i=1}^{I} \left\| \vec {v_{i}} - \vec {v_{i-1}} \right\| \end{aligned}$$

where $\vec {v_{i}}$ is the average vector velocity inside the considered area for the time frame *i*, *I* the total number of frames considered and $\Delta t$ the time difference (in seconds) between two consecutive frames. For each trial the velocity fluctuation on the left and right areas have been computed and the average between both has been taken as the representative value for that specific trial.

#### Alignment parameter fluctuation

In addition to the velocity fluctuation another measure has been taken to account for changes in velocity, the so-called “alignment parameter”. This measure had been already introduced in^26^, but in that case the whole room had been accounted and it was not used in the frame of a statistical analysis comparing several experiments.

**Figure 7:**
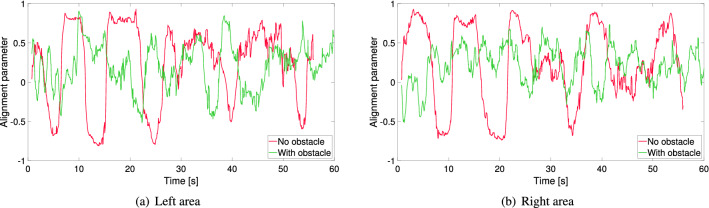
Evolution of the alignment parameter in the regions of Fig. 5 for two selected experiments with the army. Both experiments had a high level of competitiveness and the distance of the obstacle (when employed) was 60 cm.

The alignment parameter for a given instant (frame) *i* is defined as:

2 $$\begin{aligned} \phi _d (i) = \frac{1}{N} \left\| \displaystyle \sum _{n=1}^{N} \frac{\vec {v_i^n} \cdot \vec {u_i^n}}{\left. \vec {v_i^n} \right. } \right\| \end{aligned}$$

where $\vec {v_i^n}$ is the vector speed of pedestrian *n*, $\vec {u_i^n}$ its desired direction of motion (which is assumed to be towards the door) and *N* the total number of of participants in the area considered. When the crowd moves in the azimuthal direction in respect to the location of the door the alignment parameter becomes 0. Organized movements toward the door will result in a value close to 1 and values close to −1 refer to a radial motion away from the exit.

A representative evolution of the alignment parameter for two trials with and without the obstacle is shown in Fig. 7. As it can be seen, both on the left and the right side, the presence of the obstacle contributes to generally stabilize the motion. Without the obstacle, moments of collective motion toward the exit are followed by sudden changes toward the opposite direction.

In each trial the standard deviation of the alignment parameter has been taken for the left and right side of the obstacle. The mean between those values has been taken as a measure for the capacity of the crowd to move in a stable and collectively organized way in a single trial.

#### Mean flow rate at the exit

Finally, to complete the list of bottleneck-specific measures, the mean (or average) flow rate at the exit also needs to be considered. This is simply defined as the number of people crossing the door per second and can be calculated as the number of participants divided by the total evacuation time (i.e. the time between the first and the last participants walking through the door). Flow rate is one of the most commonly used quantities to assess evacuation efficiency and it is also an important value used in building codes^43^. Although an extensive analysis relative on the flow rate for the considered experiments has been already carried out in previous works^26,27^, its relation with other universal crowd measures was never studied and we judged therefore necessary reconsidering it in this work.

### Crowd pressure on the walls

Finally, in an attempt to further evaluate the conditions inside the crowd in each experimental setup, we also developed a method to estimate the pressure felt by participants close to the exit by only relying on tracking data. As said earlier, in the soldier experiments, a pressure sensor was attached to the doorjamb actually measuring the pressure exerted by the crowd. However, it is generally not possible to use such a sensor to monitor crowd conditions in a real environment, since it would too costly to install such sensors in emergency exits. For this reason, we tried to understand if it is possible to estimate the crowd contact pressure (at least at the exit) only based on people positions (which could be obtainable by a surveillance camera).

**Figure 8:**
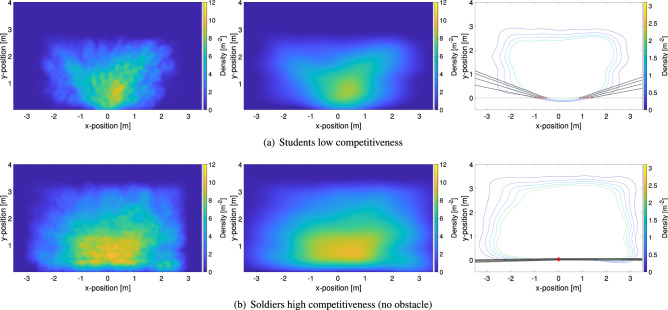
Analytical approach used to determine the angle of the crowd at the exit and potentially estimate crowd pressure. Left: raw density maps obtained using the disk method. Center: fitted density profile. Right: isometric density lines and angle formed with the wall at the exit. Two selected experimental trials were used to generate these results. Note that density profile presented here is obtained using the disk method and values can therefore be slightly different from the Voronoi approach.

We may assume that when people push toward a wall the distribution of density is parallel to the direction of the wall, since people closer to wall are under the influence of a bigger pressure. From the density profiles provided on the left of Fig. 8 and relative to a low and an high competitiveness evacuations, it can be seen that the density of the crowd shows a very different shape: a sort of “V” profile for low competitiveness and a more flat distribution in the competitive case. Although in non-competitive conditions the shape of the density distribution depends on the way people choose to line up^44^ and the specific geometry of the room^45^, in the competitive case we may assume it will be the flatter the more people push toward the exit. Thus, the angle formed by the density profile at the exit could allow estimating the pressure inside the crowd.

The hereafter defined “crowd angle” (i.e. the angle of the density profile) has been therefore computed using the following procedure also illustrated in Fig. 8 from left to right.

1. A density profile has been computed for each trial. This time the so-called disk method^46^ has been used to compute density. The experimental area has been discretized into a fine mesh with a cell width of 5 cm. In each frame a disk 25 cm in radius (roughly corresponding to the surface of the human body) has been set in the position of each participant and all the cells contained with the surface of the disk have been sequentially increased by one. This procedure was repeated in all frames for all participants and the total counts in each cell where later normalized using the number of frames and the surface of the disk. This procedure was preferred to the Voronoi approach described earlier because it is accurate also close to the exit, which is the most important area for angle calculation (as already mentioned, Voronoi cells are not accurately computed in front of the exit due to missing trajectories past the door). On the other side, the disc method is largely inaccurate around the obstacle, but that area is irrelevant in computing the angle with the wall.
2. The obtained density distribution was later fitted using a (surface) local linear regression to obtain a smoother profile. Smoothing parameter was chosen taking a compromise between smoothness of the generated profile and ability to maintain qualitative and quantitative features.
3. Using the smoothed density profile isometric density lines where computed in $0.5 \, {\hbox {m}}^{-2}$ steps from a density of $0.1 \, {\hbox {m}}^{-2}$ to $3.1 \, {\hbox {m}}^{-2}$. In the case of the obstacle, two sets of isometric lines are generated: one enclosing the external boundaries of the crowd (walls and unoccupied regions) and another one concentric to the obstacle (where density is zero per definition). In the angle analysis, only the external isometric lines are considered and the ones starting from the obstacle are neglected. Also, in addition to the ones relative to the obstacle, isometric lines being too course (thus not allowing precise angle calculation) were later removed.
4. The angle of each isometric line was computed distinguishing the case where isometric lines cross the wall or not. When isometric lines intersected the wall the steepness was defined taking the intersecting point with it and a second point lying 5% away along the isometric curve. If isometric lines were contained within the room, the center of the exit ($x = 0 \, \hbox {m}$) was taken as the reference point (with the second one taken again at a 5% distance along the curve).
5. Finally, the “crowd angle” was computed taking the average angle between both sets of curves. For a distribution perfectly parallel to the wall an angle of one radian will be obtained.

## Results and discussion

In this section, the main results of this work are presented. As a general rule we will follow the same order used in introducing the methods, with universal crowd measurements coming first and bottleneck specific presented later. In a concluding part we will consider whether there is a correlation between the two types of quantities and which universal crowd measures is the most appropriate to evaluate the evacuation experiments analyzed here.

### Universal crowd measures

In this part, the distribution of density, congestion level and crowd danger is shown for each experimental condition using colored maps highlighting spatial variations. All local distributions presented here are the average relative to each experimental condition. The experiments performed with students are presented first, with the ones relative to the army following in the presentation.

**Figure 9:**
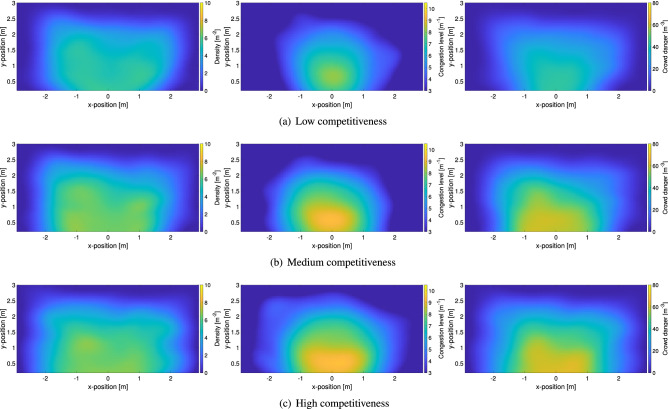
Spatial distribution for relevant crowd properties in relation with the level of competitiveness. Students’ experiments are presented here (hence all did not have the obstacle). Density maps (using Voronoi cells) are provided on the left, congestion level in the center and crowd danger on the right side.

Figure 9 shows the distribution of the different crowd quantities in the experiments with the students, where only the level of competitiveness is varied. It is clearly seen that all quantities increase in relation with the level of competitiveness, but a larger increase is seen when competitiveness is changed from low to medium, with a smaller variation when passing from medium to high.

If density is considered, it can be noted that its distribution changes with competitiveness. At low levels a sort of “V” shape is observed with locations being far or directly in front of the exit having a slightly lower density. However, at medium and high levels of competitiveness a more concentric shape is observed (the reason why a semicircle is not observed could be related with the fact that tracked area is limited to about 2.5 m in the *y*-direction). In general it can be even seen that quite high density values are reached, with regions close to the exit having a maximum density of around $7 \, {\hbox {m}}^{-2}$; quite a considerable value considering this is the average over the whole evacuation process.

A first consideration in regard to the congestion level is that results are generally not so different from density. Although this might seems obvious since the same experiment is considered, it should be reminded that density is actually computed based on pedestrians’ position, while the congestion level ultimately relies on the velocity vector field which does not depend on people’s positions (although here trajectories were used to generate it). In short, this shows that under packed conditions when commercial tracking systems typically fails^47^ (consider that people usually do not wear colored caps like in these experiments) the congestion level allows getting a rough estimation of density from the crowds’ vector field, which, on the contrary, could be easily obtained in packed conditions using an optical flow approach^48^.

When results are considered more in detail, it can be noted that the distribution of density and congestion level is indeed different. While density is generally high for locations close to the exit, the maximum of congestion is clearly localized right in front of the exit. As previous research already found^29,30^ under non-competitive conditions the maximum congestion is found slightly in front of the exit (at a distance similar to the exit width), but the maximum moves right at the position of the exit when the level of competitiveness is increased. This can be understood with the fact that under non-competitive conditions people try to find a sort of arrangement before leaving, thus moving in a sort of organized way through the exit and in front of it. However, under competitive conditions, most unorganized interactions occur close to the exit, which is where the maximum is seen.

Finally, the crowd danger, being the product of density and congestion level, shows a combination of both distributions’ features. A peculiarity of the crowd danger lies in the strong decrease observed when moving away from the exit, possibly showing that the most dangerous area at bottlenecks is limited to a few meters from the exit. The maximum reached for crowd danger is in line with previous studies which considered people moving randomly inside a packed room^29^. However, as a term of comparison, it should be noted that values of $70 \, {\hbox {m}}^{-3}$ are typically reached at a density of around $9 \, {\hbox {m}}^{-2}$ in unidirectional pedestrian streams^29^, thus showing that bottlenecks are already dangerous at lower densities (here about $7 \, {\hbox {m}}^{-2}$).

**Figure 10:**
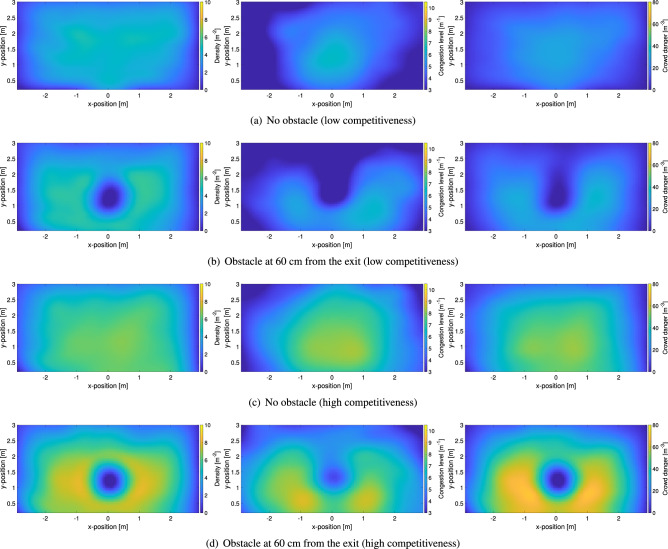
Spatial distribution for relevant crowd properties in relation with the level of competitiveness and the presence of an obstacle. Soldiers’ experiments are presented here and, when employed, the location of the obstacle is clearly recognizable. Density maps are provided on the left, congestion level in the center and crowd danger on the right side.

Figure 10 presents the spatial distributions relative to the experiments with soldiers. In this case, both the effect of competitiveness and obstacle presence are investigated. When the cases without obstacle are examined, it can be seen that results are in line with the ones obtained with the students: density and congestion level show similar profiles with the congestion level having a more concentric shape in which its maximum moves towards the exit with an increase in competitiveness. Maximum values are also similar, although, quite surprisingly congestion level was higher in the experiments with students, despite the fact physical interactions were much less important. This partially shows one of the limitations of the congestion level, which considers smoothness of motion but cannot account for the actual pressure felt by participants (or their concentration, being also one reason why the crowd danger was defined).

We can now consider the most relevant part of this work, namely the obstacle effect. At low levels of competitiveness differences between the case with and without the obstacle are minimal and mostly limited to the region behind the obstacle (and the center of the obstacle itself, but this is simply because people cannot stand there). Both density and congestion level are lower in the area behind the obstacle, both contributing in a reduction of the crowd danger.

However, when the competitive case is considered, a remarkable change is created by the presence of the obstacle. Density, which was roughly equally distributed within the room without obstacle, is much higher and peaking around the obstacle when this is employed. Areas behind and also in front of the obstacle have generally lower densities compared to lateral areas (for areas in front of the exit, this may also because of the limitations given by the Voronoi method as discussed earlier on). When it comes to the congestion level, a qualitative change is observed when the obstacle is used. Congestion, which had a maximum centered in front of the exit without the obstacle, is now peaking in the regions between the obstacle and the wall. In short, it seems like the obstacle has created two bottlenecks between its location and the wall instead of reducing overall congestion.

An additional remark can be made on congestion level’s results, in which it is observed that overall comparable values are recorded in competitive conditions with and without the obstacle. This seems to contradict with the representation of Fig. 6, where velocity fluctuations are noticeably larger without the obstacle. Here, we would like to remind that congestion level is computed on a microscopic scale, in which differential analysis is performed on a mesh 0.2 m in size. On the other side, velocity fluctuations were computed taking an average over a large area (2 m in side length). As a consequence, what could be seen as a stable or “organized” motion from a macroscopic perspective (velocity fluctuations) can indeed be more chaotic on a microscopic scale (congestion level), thus leading to seemingly contradicting results.

Crowd danger again reflects both features found in density and congestion level and regions around the obstacle and in particular between the obstacle and the wall seems to be the most dangerous ones. Interestingly, the crowd danger in front the exit is lower compared to neighboring regions, possibly implying the obstacle indeed allows “protecting” people lying between its location and the exit. Values for crowd danger are now almost 10% higher compared to the experiments with students, which shows that more extreme conditions were recreated in the case of soldiers.

**Figure 11:**
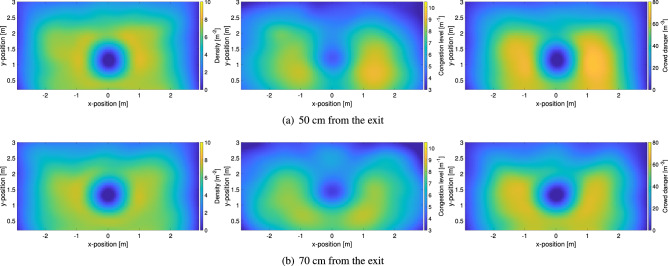
Spatial distribution for relevant crowd properties in relation with the distance of the obstacle to the exit. The case for 60 cm is reported in Fig. 10d and is not repeated here for the sake of brevity.

Some of the initial observations performed while considering the presence of obstacle in competitive conditions are confirmed when the effect of the obstacle position is examined, as presented in Fig. 11. Density seems to be generally unrelated with the distance of the obstacle to the door, with similar profiles for the three cases considered (the 60 cm distance was already provided in Fig. 10d). However, it is now clearer that the obstacle contributes in increasing the congestion in the two “channels” formed with the wall. In particular, at 50 cm, when the distance to the wall is the smallest, two highly congested regions appear at both sides of the obstacle, close to the wall. On the other side, at 70 cm (the largest distance), the highly congested region appears to be more equally distributed around the obstacle partially covering also the region in front of the exit. This characteristic appears to be beneficial in terms of crowd danger, with the 70 cm configuration showing the lower values among the three cases considered. Details on (local) variations caused by the presence of the obstacle will be discussed later and are also given in the supplementary material.

#### Summary

So far the distribution of the different quantities has been discussed mostly from a qualitative perspective, without paying too much attention on the actual values and their statistical significance. In this concluding part we wish to compare the various experimental conditions from a statistical point of view and try to summarize how the level of competitiveness, the presence of the obstacle and its distance affect overall crowd dynamics.

On this purpose an analysis of variance (ANOVA) test has been performed using all trials available for each condition and relating experimental variables with crowd quantities. For each trial the average over the whole surface is taken (non-smoothed profiles are used), with results for this analysis reported in Table 2.

**Table 2:** Analysis of variance (ANOVA) test for the three universal crowd quantities in relation with the experimental conditions. Statistical significance of each condition (for example level of competitiveness: low, medium and high; or obstacle position: 50 cm, 60 cm and 70 cm) has been computed taking all trials belonging to a specific class of experiments. Values given in bold are statistically significant on the 5% level of confidence and indicate therefore a correlation between experimental condition and presented quantity.

ANOVA result	Competitiveness (student dataset)	Presence of obstacle (soldier dataset)		Obstacle position (soldier dataset)
		Low competitiveness	High competitiveness	
Density	$\mathbf{p } < \mathbf{0 }.\mathbf{001 }, \mathbf{F(2,31) } = \mathbf{28 }.\mathbf{54 }$	p = 0.385, F(1,5) = 0.95	$\mathbf{p } = \mathbf{0 }.\mathbf{030 }, \mathbf{F(1,12) } = \mathbf{6 }.\mathbf{24 }$	p = 0.306, F(2,17) = 1.28
Congestion level	$\mathbf{p } < \mathbf{0 }.\mathbf{035 }, \mathbf{F(2,31) } = \mathbf{57 }.\mathbf{27 }$	p = 0.803, F(1,5) = 0.07	p = 0.328, F(1,12) = 1.05	p = 0.627, F(2,17) = 0.48
Crowd danger	$\mathbf{p } < \mathbf{0 }.\mathbf{001 }, \mathbf{F(2,31) } = \mathbf{57 }.\mathbf{01 }$	p = 0.451, F(1,5) = 0.70	$\mathbf{p } = \mathbf{0 }.\mathbf{020 }, \mathbf{F(1,12) } = \mathbf{7 }.\mathbf{46 }$	p = 0.311, F(2,17) = 1.26

As it was already clearly seen in the distributions maps, it is statistically confirmed that level of competitiveness has a significant role in regard to all crowd quantities, namely making them higher in competitive conditions. It is also confirmed that, statistically speaking, when the whole room is considered, the distance of the obstacle is not influencing crowd dynamics in a significant manner, although we have seen that local variations do occur. This may also be related to the little variation in the distances taken. In fact, it is seen that under competitive conditions, when the obstacle is removed (thus corresponding to an infinite distance), a significant difference is seen in density and crowd danger (both decrease), while no significant change occurs to the congestion level. However, no significant relationship between all crowd quantities and the presence of obstacle is seen in non-competitive conditions, showing that, at least in which concerns the quantities considered here the obstacle does not seem having a beneficial effect.

**Figure 12:**
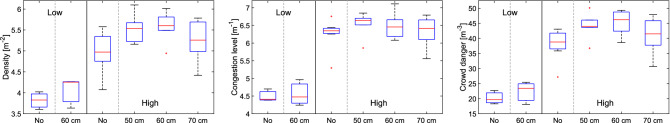
Statistical values for density, congestion level and crowd danger in the whole experimental room. In the graphs “Low” and “High” stand for the level of competitiveness with distance to the obstacle provided below (“No” meaning no obstacle). Median value is given as a red line inside the box showing the 25th and 75th percentiles on the bottom and the top, respectively. Minimum and maximum are represented by the extrema of the whiskers with outliers represented using a “+” sign.

Figure 12 provides a closer look on the statistical analysis presented in Table 2. In Fig. 12 results relative to the experiments with students have been omitted as it is already clear that all quantities increase in competitive conditions. Again, in all the cases the average over the whole room is taken by using the raw data for each map (i.e. before performing the smoothing operation).

An interesting feature which can be noted in the three quantities is that (under competitive conditions) the role of the obstacle seems to quickly diminish with the distance. In fact, most median values for the 70 cm case are similar to the values reported without the obstacle. This may confirm that although distances chosen here did not lead to statistically significant differences, a change of 20 cm can already lead to modifications in crowd dynamics as it was also qualitatively observed in the distribution maps.

To conclude this part, it should be remarked that no particular features have been observed in regard to temporal evolution of crowd quantities during the evacuations. Nonetheless, temporal profiles are reported in the supplementary material for completeness.

### Bottleneck specific measures

We now wish to consider the specific quantities designed to investigate the presence and extent of low-frequency crowd oscillations and general instabilities not captured by the measures presented above. We will follow the same order of presentation used in describing the methods. Mainly qualitative aspects will be discussed first for each quantity and later we will summarize all of them from a statistical perspective.

**Figure 13:**
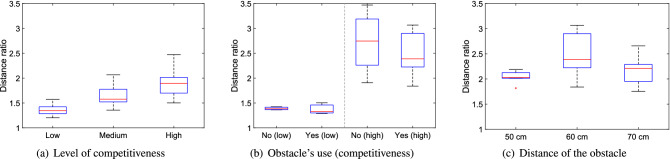
Distance ratio under different conditions. Low, medium and high stand for the level of competitiveness, yes/no is relative to the presence of the obstacle and the distances provided are relative to the ones between the obstacle and the exit.

Results for the so-called distance ratio are presented in Fig. 13. As it can be seen the level of competitiveness clearly contributes in increasing the relative distance traveled by participants. The relationship is clear both in the experiments with students (see Fig. 13a) and the ones with soldiers (see Fig. 13b for the cases without obstacle), where the change in distance ratio is even bigger, possibly due to the fact that soldiers were allowed to push to a greater extent and also due to the larger participants’ number in the soldiers case (almost double compared to students).

When the presence of the obstacle is considered (Fig. 13b) a rather interesting result is obtained: despite the large variations, distance ratio is generally smaller when the obstacle was employed, especially in the competitive conditions. This is rather counter-intuitive, since the obstacle would make relative traveled distances longer. So, this result can only be explained assuming that the obstacle allows to stabilize crowd motion, thus damping large fluctuations (as it was originally seen in Fig. 6).

Concerning the distance of the obstacle, there is no clear relationship with the distance ratio, possibly implying that, from this point of view, the investigated distances are too close to each others’.

**Figure 14:**
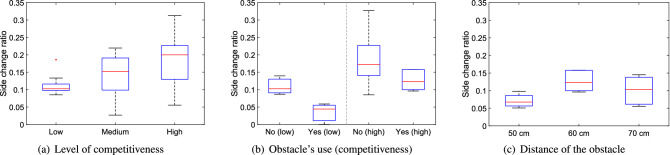
Side change ratio under different conditions. Low, medium and high stand for the level of competitiveness, yes/no is relative to the presence of the obstacle and the distances provided are relative to the ones between the obstacle and the exit.

When the side change ratio (the amount of participants starting on one side but leaving from the other) is considered, as reported in Fig. 14, a similar behavior to the distance ratio is seen. However, a significant difference from the previous measure is seen when comparing the cases with/without obstacle. In fact, the ratio of people who changed side is reduced by the presence of the obstacle and is particularly small at low competitiveness (with zero reached in one trial, a unique occurrence throughout all experiments). This implies that the obstacle is beneficial in channeling people to the exit and this seems particularly effective at low competitiveness, possibly because in non-competitive conditions people are able to walk at their own willingness and are not moved around by collective crowd waves. Again, when obstacle distance is considered, no clear effect is seen even on the side change ratio, but the three results (when compared to the case without obstacle) confirm the capability of the obstacle to funnel pedestrian motion.

**Figure 15:**
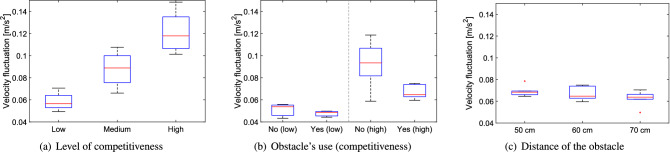
Velocity fluctuation under different conditions. Low, medium and high stand for the level of competitiveness, yes/no is relative to the presence of the obstacle and the distances provided are relative to the ones between the obstacle and the exit.

Next, we wish to consider the velocity oscillation (see Fig. 15) which allows to directly investigate the extent of collective crowd waves, a possible explanations for the results observed so far. As for the previous quantities, a linear increase is seen for the level of competitiveness, showing that instabilities build up as people become competitive. However, when the obstacle effect is considered, this time the biggest change is seen under highly competitive conditions, where the obstacle is confirmed having a stabilizing effect, thus greatly reducing long-term oscillations. When the experiments with the soldiers are considered it is clearly seen that, independently on the distance, every time the obstacle was employed, velocity oscillations were smaller compared to the case without obstacle (although, again distance seems having little influence).

When the experiments with students and soldiers are compared it can be noted that students had higher values for velocity fluctuations. This can be explained considering that participants’ number was almost half in the case with students and therefore oscillations could not be damped by the presence of other people around.

**Figure 16:**
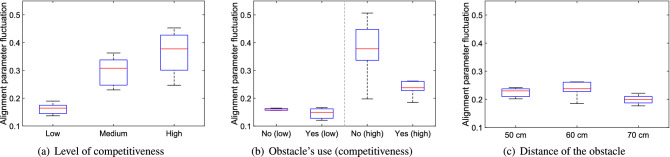
Alignment parameter fluctuation under different conditions. Low, medium and high stand for the level of competitiveness, yes/no is relative to the presence of the obstacle and the distances provided are relative to the ones between the obstacle and the exit.

The fluctuation of the alignment parameter (see Fig. 16) further confirms what discussed earlier, i.e. that the obstacle has a beneficial effect in stabilizing collective crowd waves. The difference between the case with and without obstacle in competitive conditions is even stronger when the alignment parameter is considered and a slight dependence on obstacle distance is now seen, with the 70 cm case seemingly the most stable (this result could be explained by observing that waves tend to be stronger further from the wall).

In the case of the alignment parameter, the maximum values for the experiments with students and soldiers are in agreement, possibly because absolute speed values are contributing less to the final result in the calculation of the alignment parameter.

**Figure 17:**
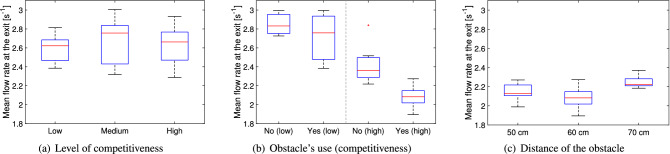
Mean flow rate at the exit under different conditions. Low, medium and high stand for the level of competitiveness, yes/no is relative to the presence of the obstacle and the distances provided are relative to the ones between the obstacle and the exit.

Finally, the mean flow rate at the exit is considered, with its results presented in Fig. 17. When the level of competitiveness is considered, it is found that, in the case of the student dataset (Fig. 17a), it did not contribute in increasing nor decreasing the flow rate, even when competition was high. However, in the case of soldiers (Fig. 17b), an apparent reduction in flow rate is observed in the competitive scenario. In this regard, we should however remind that non-competitive evacuations (without obstacle) for soldiers consisted of only three trials and a more detailed analysis on the flow rate performed in^26^ (where instantaneous flow rates are used) revealed that relation between flow rate and competitiveness is non-existing or minimal. Nonetheless, as discussed in^26^, a reason to explain the possible reduction in flow rate for competitive conditions could be related to the occurrence of clogging, i.e. long-time intervals between exiting participants being the consequence of unresolved conflicts between pedestrians gathering at the exit.

When the role of the obstacle is examined (Fig. 17b), it is found that its presence in competitive conditions would reduce the mean flow rate, thus contribute in making evacuations longer (a reduction is partially observed also in non-competitive conditions, although much less clear). This result is more difficult to explain, since, as already mentioned in the introduction, many studies found the opposite. Based on the evidence collected so far in this work, we could speculate that the high density at the exit and the absence of strong oscillations caused by the obstacle, could make it more difficult to stop long-lasting unresolved conflicts occurring at the exit. In this sense, the lower densities and the strong oscillations observed without the obstacle could help breaking strong conflicts more regularly, thus increasing flow rate at the exit. This hypothesis could be partially justified by observing that in non-competitive conditions (when conflicts are less important), differences in mean flow rate are less marked.

Concerning obstacle distance (Fig. 17c), it is found again that, at least for the cases investigated here, differences in flow rate also are not important.

#### Summary

The statistical relation between the quantities designed for the bottleneck scenario and the experimental variables is presented in Table 3 using an ANOVA test.

**Table 3:** Analysis of variance (ANOVA) test for the five measures considered to study stability of the bottleneck case in relation with the experimental variables. As for the previously presented Table 2, statistical significance of each condition has been computed taking all trials belonging to a specific class of experiments. Again, values given in bold are statistically significant on the 5% level of confidence and indicate therefore a correlation between experimental condition and presented quantity.

ANOVA result	Competitiveness (student dataset)	Presence of obstacle (soldier dataset)		Obstacle position (soldier dataset)
		Low competitiveness	High competitiveness	
Distance ratio	$\mathbf{p } < \mathbf{0 }.\mathbf{001 }, \mathbf{F(2,31) } = \mathbf{19 }.\mathbf{57 }$	p = 0.834, F(1,5) = 0.05	p = 0.411, F(1,12) = 0.73	p = 0.095, F(2,17) = 2.77
Side change ratio	$\mathbf{p } = \mathbf{0 }.\mathbf{035 }, \mathbf{F(2,31) } = \mathbf{3 }.\mathbf{76 }$	$\mathbf{p } = \mathbf{0 }.\mathbf{033 }, \mathbf{F(1,5) } = \mathbf{10 }.\mathbf{18 }$	p = 0.102, F(1,12) = 3.19	$\mathbf{p } = \mathbf{0 }.\mathbf{028 }, \mathbf{F(2,17) } = \mathbf{4 }.\mathbf{57 }$
Velocity fluctuation	$\mathbf{p } < \mathbf{0 }.\mathbf{001 }, \mathbf{F(2,31) } = \mathbf{59 }.\mathbf{66 }$	p = 0.466, F(1,5) = 0.65	$\mathbf{p } = \mathbf{0 }.\mathbf{010 }, \mathbf{F(1,12) } = \mathbf{9 }.\mathbf{71 }$	p = 0.203, F(2,17) = 1.78
Alignment parameter fluctuation	$\mathbf{p } < \mathbf{0 }.\mathbf{001 }, \mathbf{F(2,31) } = \mathbf{41 }.\mathbf{76 }$	p = 0.351, F(1,5) = 1.11	$\mathbf{p } = \mathbf{0 }.\mathbf{006 }, \mathbf{F(1,12) } = \mathbf{11 }.\mathbf{43 }$	$\mathbf{p } = \mathbf{0 }.\mathbf{027 }, \mathbf{F(2,17) } = \mathbf{4 }.\mathbf{61 }$
Mean flow rate at the exit	p = 0.604, F(2,31) = 0.51	p = 0.515, F(1,5) = 0.509	$\mathbf{p } = \mathbf{0 }.\mathbf{005 }, \mathbf{F(1,12) } = \mathbf{12 }.\mathbf{12 }$	$\mathbf{p } = \mathbf{0 }.\mathbf{036 }, \mathbf{F(2,17) } = \mathbf{4 }.\mathbf{18 }$

As it was the case for universal crowd quantities, also for the specifically designed ones, a clear dependence with the level of competitiveness (as observed in the student dataset) is seen for most of the quantities (also here increasing in higher competitive scenarios), except for the mean flow rate. However, if competitiveness is compared in the soldier dataset (considering only experiments without the obstacle), a significant difference in mean flow rate was found (p = 0.013, F(1,9) = 10.26), but, given the limited number of trials, more data would be needed for a consistent conclusion.

When the role of the obstacle is further considered, it can be concluded that it has a channeling effect at low competitiveness (as shown by the side change ratio) and a stabilizing effect at high competitiveness (as shown by the velocity and alignment parameter fluctuation). These conclusions would be generally in line with the studies performed so far by other researchers, where the positive effect was mostly observed at low or non-competitive conditions and for small crowds^23^. As already discussed, the increase of density at the exit and the absence of oscillation waves in competitive conditions with the obstacle could explain the negative change observed in the mean flow rate.

Distance ratio had no significant relation with the presence of the obstacle, but, as already discussed, this should indicate that the obstacle did not make participants walking longer, which would on the contrary show that it had a role in stabilizing overall motion.

Concerning the distance of the obstacle, it is quite difficult to summarize its relevance. While some quantities showed a significant relation with it, there was not always a clear trend, like in the case of the side change ratio. It is more likely that obstacle’s distance (at least for the range considered) has consequences on the local level (as discussed earlier) and changes on the global scale are limited or insignificant.

### Correlation between universal and specific crowd quantities

After having considered universal and scenario-specific crowd quantities we may now consider whether there is a correlation between these two sets and which universal quantity also allows to accurately examine the bottleneck case. Table 4 presents the correlation coefficient^49^ between both sets of quantities taking all experimental trials as variables to test their linear dependance.

**Table 4:** Correlation coefficient between universal and scenario-specific crowd quantities for the bottleneck case. Each trial of all experiments (both students and soldiers) has been used in computing the correlation (thus a total of 63 trials were considered). An higher correlation coefficient indicates a closer linear correlation between both quantities.

Correlation coefficient	Distance ratio	Side change ratio	Velocity fluctuation	Alignment parameter fluctuation	Mean flow rate at the exit
Density	$\mathbf{0 }.\mathbf{473 } \, (\mathbf{p } < \mathbf{0 }.\mathbf{001 })$	$\mathbf{0 }.\mathbf{263 } \, (\mathbf{p } = \mathbf{0 }.\mathbf{038 })$	$\mathbf{0 }.\mathbf{509 } \, (\mathbf{p } < \mathbf{0 }.\mathbf{001 })$	$\mathbf{0 }.\mathbf{558 } \, (\mathbf{p } < \mathbf{0 }.\mathbf{001 })$	${-}\mathbf{0 }.\mathbf{340 } \, (\mathbf{p } = \mathbf{0 }.\mathbf{007 })$
Congestion level	$\mathbf{0 }.\mathbf{646 } \, (\mathbf{p } < \mathbf{0 }.\mathbf{001 })$	$\mathbf{0 }.\mathbf{341 } \, (\mathbf{p } = \mathbf{0 }.\mathbf{006 })$	$\mathbf{0 }.\mathbf{548 } \, (\mathbf{p } < \mathbf{0 }.\mathbf{001 })$	$\mathbf{0 }.\mathbf{588 } \, (\mathbf{p } < \mathbf{0 }.\mathbf{001 })$	${-}\mathbf{0 }.\mathbf{445 } \, (\mathbf{p } < \mathbf{0 }.\mathbf{001 })$
Crowd danger	$\mathbf{0 }.\mathbf{570 } \, (\mathbf{p } < \mathbf{0 }.\mathbf{001 })$	$\mathbf{0 }.\mathbf{286 } \, (\mathbf{p } = \mathbf{0 }.\mathbf{023 })$	$\mathbf{0 }.\mathbf{533 } \, (\mathbf{p } < \mathbf{0 }.\mathbf{001 })$	$\mathbf{0 }.\mathbf{579 } \, (\mathbf{p } < \mathbf{0 }.\mathbf{001 })$	${-}\mathbf{0 }.\mathbf{412 } \, (\mathbf{p } < \mathbf{0 }.\mathbf{001 })$

In general, all correlation coefficients are typically low, meaning that there is no clear correlation between a specific pair of quantities nor there is a universal measure capable to relate well with most of the bottleneck specific measures. Although the congestion level apparently shows a higher correlation with bottleneck-specific quantities, coefficients are still low and not considerably different from other quantities.

The only remarkable observation in regard to the correlation coefficients presented in Table 4 concerns the difference in sign between mean flow rate at the exit and the rest of bottleneck specific crowd quantities. Although correlation coefficients associated with flow rate are also small, the difference in sign is relevant to understand pedestrian dynamics at bottlenecks. While congestion level can be associated with a lack of capability to move smoothly and it could be natural to associate an increase in congestion level to a decrease in flow rate, the role of density is less obvious, as, apparently, more dense crowds (composed of “more” people) should produce an higher flow rate. This shows again that overall crowd stability and the capability to efficiently solve conflicts at the exit are the most important aspects in bottlenecks and therefore obstacle employment should be considered in regard to crowd conditions in order to increase evacuation efficiency and minimize egress time.

### Crowd pressure on the walls

Finally, to complete the results, we wish to consider the pressure exerted by the crowd on the doorjamb and whether it correlates with some of the tracking-based quantities.

**Figure 18:**
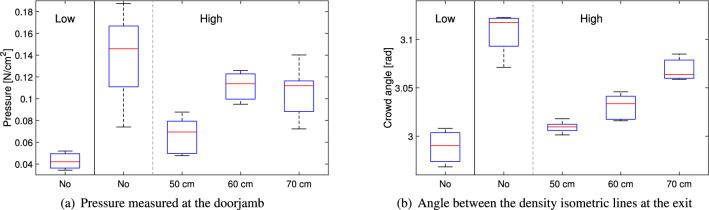
Measured and estimated pressure at the doorjamb. In the graph “Low” and “High” are relative to the level of competitiveness, with the distance of the obstacle provided below (“No” when no obstacle was employed). The low competitiveness scenario is provided for references, but only highly competitive cases should be considered valid using the isometric density lines.

Figure 18 provides a comparison between the measurements relative to the pressure sensor and the estimation performed taking the angle of the isometric density lines. Generally, a good agreement between the measured and estimated values is found (although obviously units are different) and it is further confirmed through the pressure readings that the obstacle had a positive effect in competitive conditions, although pressure was measured only in one location and little can be said for other parts of the room.

In this context, it should be noted that the non-competitive case should be regarded as a separate scenario, since estimation through the angle would be inaccurate (it depends on the willingness of the crowd to align in a particular shape) and participants’ contacts with the walls were sporadic and often accidental (people touched the sensors with the arm without being actually pressed against it; more discussion on this subject can be found in^27^). For this reason, to further evaluate the correlation between pressure measurement and its estimation, only the competitive cases have been considered.

In addition, we also considered crowd quantities obtained close to the exit (a semicircular region having a radius of 75 cm and centered at the exit was used) and checked whether they would correlate with the pressure at the doorjamb. These results for the correlative analysis are presented in Table 5.

**Table 5:** Correlation coefficient between pressure measured at the doorjamb and different crowd measures based on tracked trajectories. For quantities based on pedestrian positions an area having 75 cm of radius and centered at the exit (coordinate (0, 0)) was taken to compute the average for each condition. Note: since pressure sensors stopped for some trials or synchronization was not possible with the videos, the correlation coefficient has been computed based on each condition (considering only competitive experiments) and not for each trial as in previous analyses. Thus, in this case only four values (corresponding to the competitive cases in Fig. 18) are used in computing the correlations and this can also explain the very high p-values obtained in this analysis.

Quantity	Unit	Region considered	Correlation coefficient
Crowd angle	[rad]	Exit location (door)	0.875 (p = 0.125)
Mean flow rate	[${\hbox {s}}^{-1}$]	Exit location (door)	0.665 (p = 0.335)
Speed	[m/s]	Exit front area	−0.207 (p = 0.793)
Density	[${\hbox {m}}^{-2}$]	Exit front area	−0.646 (p = 0.354)
Congestion level	[${\hbox {m}}^{-1}$]	Exit front area	0.836 (p = 0.164)
Crowd danger	[${\hbox {m}}^{-3}$]	Exit front area	−0.051 (p = 0.949)

The crowd angle shows the best correlation, thus confirming that is possible to estimate the pressure inside the crowd, at least at the exit, in an indirect way. The congestion level similarly shows a high correlation with pressure, performing the best among the crowd quantities previously considered in the analysis and implying that it could be a good indicator for physical pressure felt by participants also for locations different from the exit. Mean flow rate at the exit shows a positive correlation with pressure, meaning that, at least in competitive conditions, a quick and usually smooth evacuation would also result in higher forces toward the doorjamb, possibly implying similar forces within the crowd. Speed shows a low and negative correlation, nonetheless providing some hints that fast and thus possibly efficient evacuations (where speed is high) could reduce the pressure at the doorjamb. However, the high p-value seen for speed indicates the correlation is among the least significant between the ones considered (all are high due to the low sample number). On the other side, density shows a very counter-intuitive result, returning a negative and yet non-negligible correlation coefficient (one would expect that a higher density should lead to a larger pressure). This could be partially due to the issues caused by the use of Voronoi at the exit (where the method becomes inaccurate), but it also shows the limitations of density as a static measure, thus not allowing to assess short-term fluctuations which could cause high pressure readings on the doorjamb. The very low correlation for crowd danger can be interpreted taking into consideration the differences in sign shown by density and congestion level (which is indeed confirmed by the extremely high p-value).

## Conclusions

The variations in crowd collective motion caused by placing a circular obstacle in front of an exit during evacuation have been studied by means of supervised experiments. Two types of experiments were considered: in the first, students were instructed to move with different levels of competitiveness and in the second professional soldiers were employed to reach extreme levels of crowdedness. Trajectories extracted from video analysis have been used in the numerical analysis which focused on classical and novel quantities employed in the frame of pedestrian dynamics. In addition, also pressure readings from a sensor installed on the doorjamb have been used for part of the analysis.

Results generally show that density and level of congestion increase with the degree of competitiveness. High competition also led to larger instabilities, both factors contributing in increasing the risk of accidents. Results in relation to the presence of the obstacle were of dual nature and related to the degree of competitiveness. Under high competition and pushy behavior, higher values for density and comparable values for congestion were found when the obstacle was used but, at the same time, lateral oscillations were reduced, thus stabilizing crowd motion. Obstacle distance from the exit was found having little relevance on crowd dynamics and mostly on a local level, specifically reducing congestion in some areas while increasing it in others. A possible reason for the small changes brought by different obstacle distances could be related to the small difference between the values chosen (only a range of 20 cm was considered, comparing with the 100 cm diameter of the obstacle).

In relation to the findings summarized above, it should be considered that the vast majority of casualties occurring during human stampede accidents are caused by external compression (leading to traumatic asphyxia), with trampling (leading to internal organ injuries) occurring to lesser extent and being the second most common cause of death^13,50–53^. According to the results of this study, the obstacle is effective in reducing the strong lateral oscillations, thus potentially decreasing the risk of trampling. However, the obstacle was also found generally increasing density (and congestion level), thus making the risk of external compression higher. Since external compression has a higher probability of resulting in death, it can be concluded that the implementation of an obstacle can be ineffective under emergency conditions or could possibly even making the situation worst.

It was also found that the location of maximum congestion is simply moved away from the door when the obstacle is moving closer to the exit. Although this may lead to a risky condition in the areas between the wall and the obstacle (forming a sort of second bottleneck), it contributed in reducing the congestion at the exit, as also confirmed by the pressure measurements (showing the lowest pressure for the smallest obstacle distance).

However, in non-emergency (but still urgent) conditions (i.e. without pushing), the obstacle did not substantially change density or levels of congestion, but allowed people to leave the exit in a more organized way by the channeling of people through the obstacle and the wall. Therefore, even though this study show risks related with the use of obstacle under emergency conditions, it is also shown that the obstacle can help improving the flow under low level of competitiveness.

As a last resort, it can be concluded that the use of obstacle is suggested only for those facilities where a clearly tested crowd management strategy is defined and control systems are available and capable of limiting pedestrian density at any moment. Airports or train stations are an example of such facilities where pedestrian density typically do not reach extremely high values and well trained personnel is prepared to manage potentially dangerous situations. However, implementing obstacles at event facilities (especially in case of temporary use) should be generally avoided, at least until more and better evidence of its beneficial role is attained.

In any case, this study shows that, besides having also benefits, the use of obstacles cannot be considered a universal almighty solution to solve congestion problems at bottlenecks. It is our belief that crowd management and design of pedestrian facilities has to be considered on a macroscopic scale where multiple elements are connected together on several levels. When included inside such a scheme, it cannot be excluded that obstacles could have a beneficial influence on pedestrian streams.

## Supplementary information

Supplementary file1
