# Supplementary material for: Systematic experimental investigation of the obstacle effect during non-competitive and extremely competitive evacuations
Source: Sci Rep. 2020 Sep 29;10:15947. doi: 10.1038/s41598-020-72733-w (PMC7525554; doi:10.1038/s41598-020-72733-w)
Supplement: Supplementary file 1 — Supplementary file1 [file 41598_2020_72733_MOESM1_ESM.pdf]

Appendix

Systematic experimental investigation of the obstacle effect during non-competitive and extremely competitive evacuations

Claudio Feliciani, Iker Zuriguel, Angel Garcimartín, Diego Maza, and Katsuhiro Nishinari

Effect of the obstacle

While commenting the results relative to the distribution of crowd quantities in regard to the distance of the obstacle, we noticed that an obstacle too close to the door (50 cm in this case) led to a creation of two highly congested regions between the obstacle and the wall. On the other side, when the distance was set at 70 cm, a more uniform distribution of the congestion level was seen around the obstacle. In this appendix we wish to investigate this aspect further.

On this purpose, two regions have been considered: a semicircle of 75 cm in radius and centered in the middle of the exit and a set of mirrored circles of 50 cm in radius lying 1 m from the exit's center in the x-axis and centered between the obstacle center-line and the wall in the y-direction (55 cm was used in the case no obstacle was used). Both regions are indicated in the left side of Figure 1. In both regions the three crowd quantities considered in this work have been computed for the experiments with soldiers under competitive conditions.

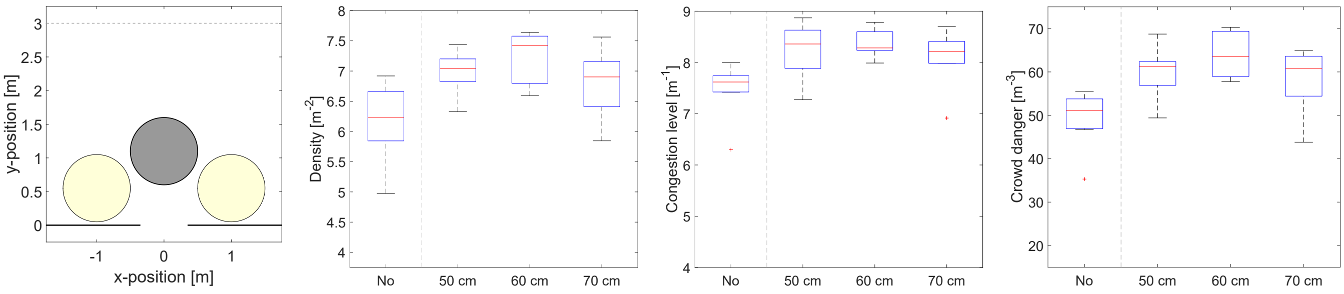

(a) Sides of the exit (between obstacle and wall)

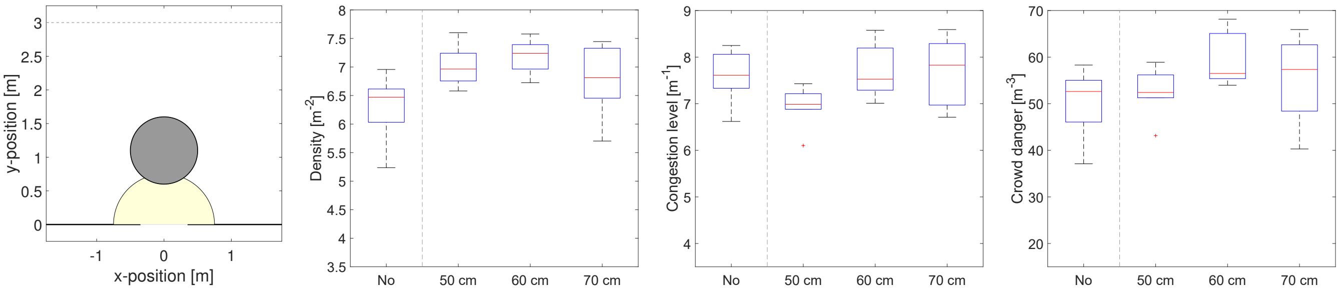

(b) Exit front

Figure 1. Relevant statistical values for density, congestion level and crowd danger in specific locations of the room and for different experimental configurations. All experimental trials for a specific conditions have been taken and results have been computed considering the non-smoothed (raw) distribution profiles. Obstacle distance for the illustrative example on the left is set at 60 cm.

Results presented in the right side of Figure 1 show that while density is not particularly affected by the distance of the obstacle (or at least not in a clear pattern), congestion level shows a partially different behavior. Although differences are minimal (and statistically insignificant) it is qualitatively confirmed that while a reduction in congestion level is observed close to the exit when the obstacle is moved toward it, an opposite behavior in seen in the regions between the obstacle and the wall. In other words, it seems congestion is not globally reduced, but locally

shifted from a location to another as obstacle distance is varied. Regardless on the distance, it is also observed that crowd danger in front of the exit is unaffected by the presence of the obstacle, but the same quantity increases for locations close to the wall.

Spatial and temporal profiles

In all the discussions so far the evacuation process has been considered as a whole, without analyzing the changes occurring from the beginning to the end. In this appendix we wish to present the results relative to the temporal evolution of the three crowd quantities considered in different areas of the room.

Analytical approach

On this purpose, each trial was divided into five equally long stages (in respect to time) from the first to the last participant walking through the exit. Using the same methods presented in the main text, density, congestion level and crowd danger were computed for each frame and, later, the average for each time-stage was computed using all frames available. As a last step, the maps relative to a single stage and for a particular experimental condition were obtained by averaging over all trials.

With the procedure presented above we have been therefore able to obtain single maps for each condition and for each stage clearly showing the location of the maximum and the minimum for each quantity within the room. However, presenting all maps would make a comparison difficult and would take unnecessary space. As a consequence we decided to focus on two locations shown in Figure 2 which we considered as the most relevant to understand how the obstacle affects pedestrian dynamics (the regions considered here are slightly different from the ones used while discussing obstacle distance because here we want to analyze evacuation as a whole, without focusing on specific aspects).

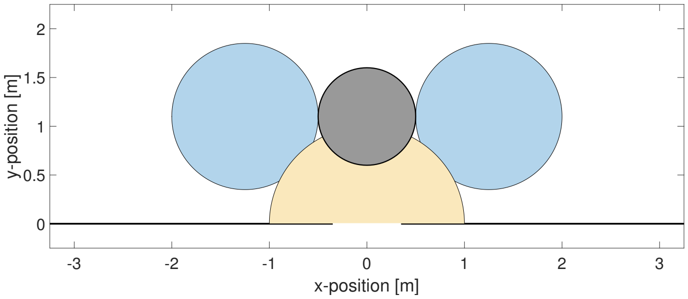

Figure 2. Areas used to determine local evolution of crowd quantities. Areas on the side of the obstacle are given in blue, the region covering the exit is given in ochre and the obstacle is given in gray. In this example the obstacle is located at 60 cm from the exit. The regions on the obstacle's side and the one on the exit, partially overlap.

More specifically, the two regions selected to perform more detailed analysis were defined as follows:

1. The region close to the exit. In this case a semicircle with a radius of 1.0 m was chosen, centered at the exit. When the obstacle was employed, part of the region was covered by the obstacle as shown in Figure 2. Owning to the fact that several stages are considered here a region slightly larger than the one typically considered for the exit front is necessary to avoid having too few data in each stage, thus leading to large variance in the results making their interpretation difficult.

2. Two circular side regions having a diameter of 1.5 m (thus having approximately the same area of the exit region). Both areas were centered at the y-axis of the obstacle (using 1.1 m in the no-obstacle condition) and were touching it on the x-axis.

Using the maps generated during each of the five evacuation stages, the average value for the three crowd quantities has been computed in each region, with both sides of the obstacle considered together (by taking their

average, owning to the symmetrical nature of the experiment). Combining all trials, average and standard deviation were computed for each condition in all locations and during all stages.

Results

In presenting the results we will follow the typical order employed so far, starting with the level of competitiveness first, considering the presence of the obstacle later and concluding by examining the effect of the distance of the obstacle from the exit.

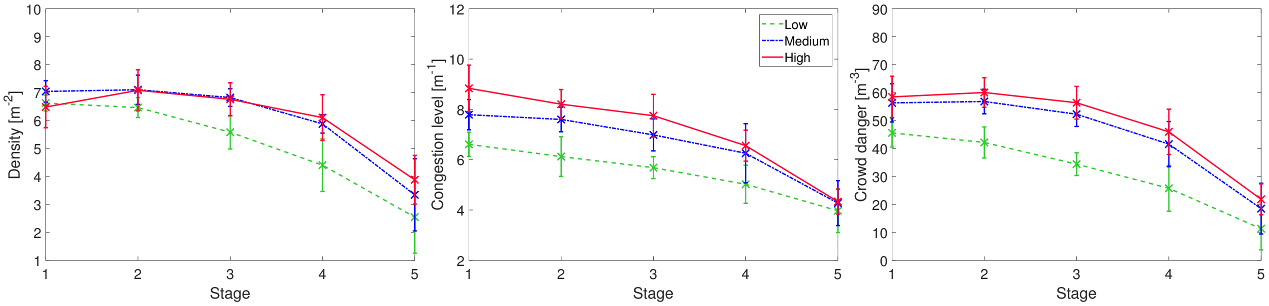

(a) Obstacle side

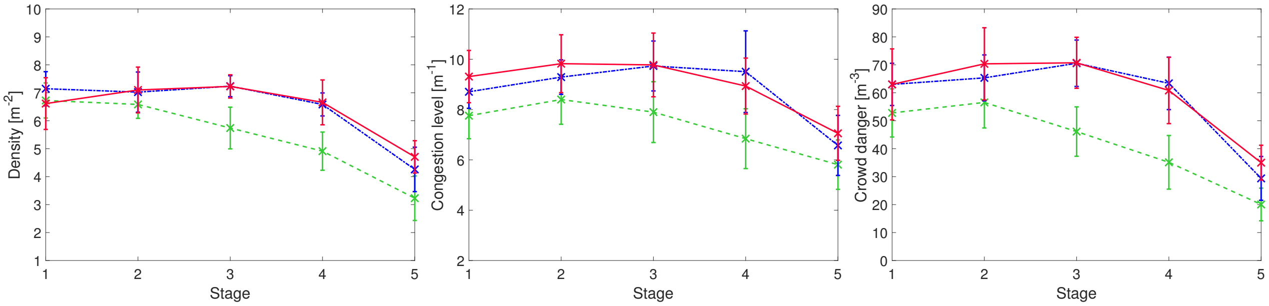

(b) Exit front

Figure 3. Temporal evolution of density, congestion level and crowd danger in relation with the level of competitiveness (thus only experiments with the students are considered here). The regions relative to the different locations are provided in Figure 2.

Figure 3 presents the result relative to the level of competitiveness. Concerning the temporal dynamics it is interesting to notice that although density has almost the same evolution in front of the exit and in the side regions, the congestion level shows a slightly different profile.

In both locations density remains roughly constant until half of the evacuation is completed to later quickly drop as many participants already left the room. Only a slight difference is noticed between the exit and the obstacle's side around stages 2 and 3, which could be easily related to the fact that participants' number start diminishing earlier in the outer parts of the crowd compared to the regions close to the exit. Also, in the case of density, medium and high competitiveness levels have almost the same values in density thorough the evacuations (a feature which was already observed in the distribution maps).

When it comes to the congestion level, it is however seen that a monotonic decrease is observed at the side of the obstacle, but a profile more similar to the one of density is seen at the exit. It is also remarkable to notice that although the three levels of competitiveness are clearly distinguished at the obstacle's sides, medium and high competitiveness are almost identical in front of the exit. This could be explained with the fact that, although participants were able to reduce physical contact when far from the exit, physical interactions became inevitable close to the exit, thus vanishing the effectiveness of the instructions. This also shows that it is not always easy to define level of competitiveness in terms of instructions, since even moderate level of competitiveness would lead to physical interactions where space among participants is limited.

Crowd danger, being a combination of congestion level and density, typically follows the pattern found in both measures. An important characteristic revealed by the temporal evolution of crowd danger concerns its maximum, which is found around stage 2 and 3 at the exit, thus at the early stages of evacuation. This shows that maintaining order especially in the initial stages of evacuation is important for people safety and partially found confirmation in the low competitiveness case, where under more orderly conditions a more linear decrease is observed.

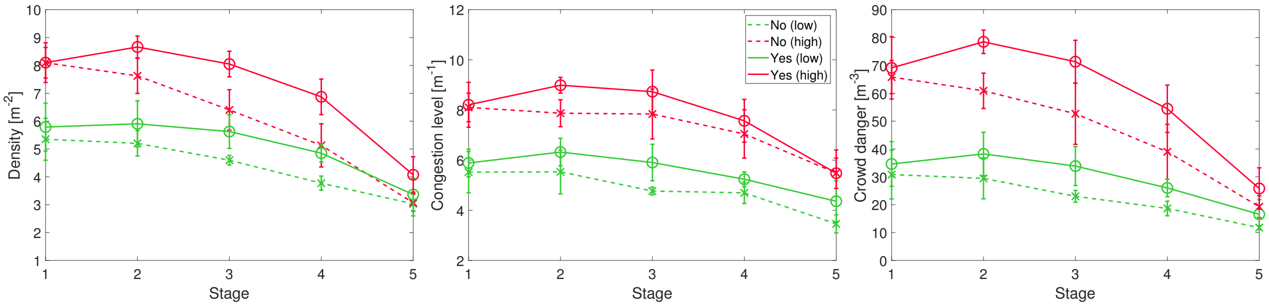

(a) Obstacle side

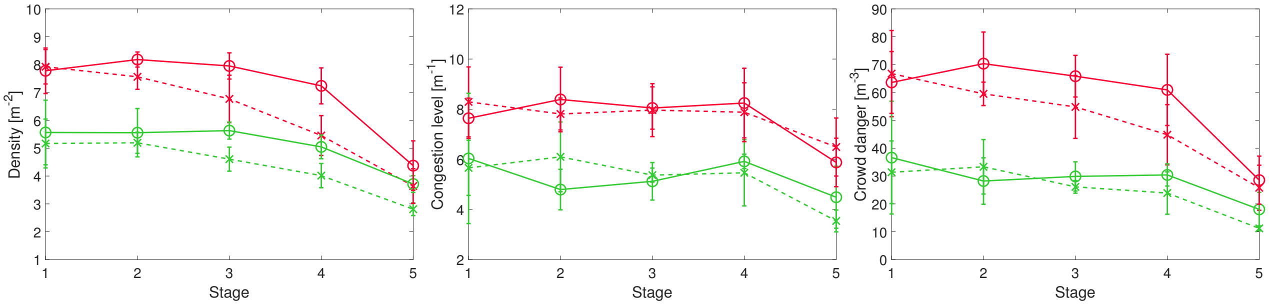

(b) Exit front

Figure 4. Temporal evolution of density, congestion level and crowd danger in relation with competitiveness level and the presence of an obstacle. When employed, distance of the obstacle was 60 cm for both level of competitiveness. The regions relative to the different locations are provided in Figure 2.

Figure 4 shows the temporal evolutions for the experiments relative to the army, in which both the level of competitiveness and the presence of the obstacle are presented. Concerning the density, similar results as for the previous case are observed, noting that density evolution is very similar for both locations considered. As it was already seen in the distribution maps, density is actually increased by the presence of the obstacle in the competitive case, but the color scale did not allow a clear distinction for the non-competitive case. In this representation it is now clearly visible that the presence of the obstacle increases density both on its sides and at the exit with this occurring independently on the level of competitiveness.

When congestion level is considered, a different profile is seen again for the exit and the sides of the obstacle. Quite interestingly, the presence of the obstacle does not seem to influence the congestion at the exit and, in the case of the non-competitive condition, at certain stages it can be observed that the obstacle actually contributes in increasing congestion. This could be related to the fact that the region considered here for the exit front partially covers the area between the obstacle and the wall, which was found having a larger congestion when the obstacle was employed.

However, when the combination of congestion level and density is considered thorough the crowd danger, it is seen that the experiments with soldiers reached higher values, indicating that reached conditions were more extreme compared to the students' experiments. Although roughly 70 m^{-3} was reached in the case of students, crowd danger went close to 80 m^{-3} for soldiers. In general, the dynamics of crowd danger during the evacuations confirms that benefits of the obstacle are limited and mostly restricted to the exit and under non-competitive conditions.

Finally, we now wish to consider the distance of obstacle during the five stages of evacuation, with the results

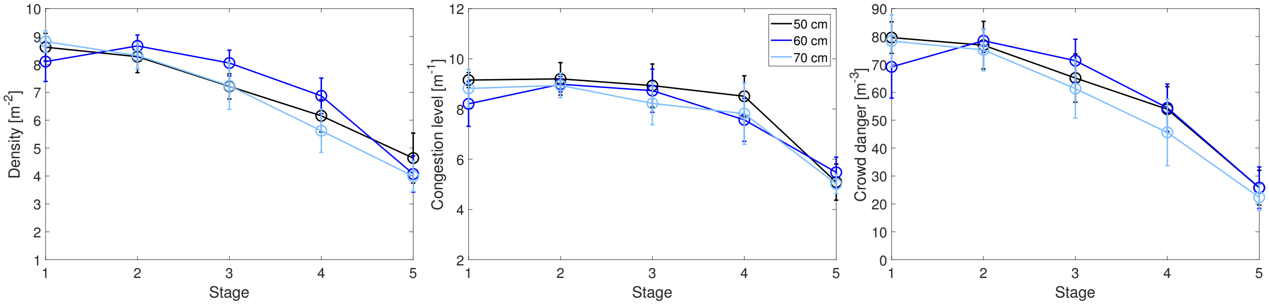

(a) Obstacle side

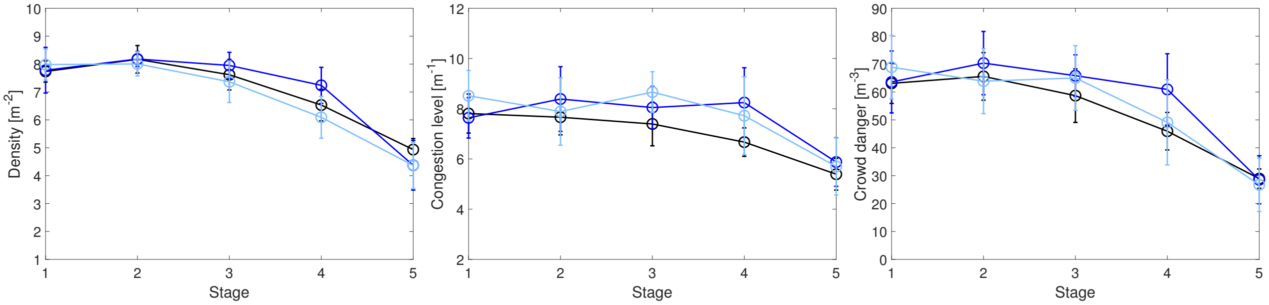

(b) Exit front

Figure 5. Temporal evolution of different crowd quantities in relation with the distance of the obstacle to the exit. The regions relative to the different locations are provided in Figure 2.

presented in Figure 5. As already discussed for most of the quantities and representations considered so far, it is difficult to find any distinguishing effect given by the distance of the obstacle from the exit, except from the local variation discussed in the previous appendix. Those observations on local variations are partially reflected in the congestion level close to the exit, where it is seen that a general reduction is observed for the 50 cm case, indicating that a closer obstacle allow to create a more smooth motion of people close to the exit (but was also shown to shift the maximum congestion elsewhere as it is partially seen by the relatively high values for the same case at the sides of the obstacle).

In this regard, it could be generally concluded that the small range chosen (20 cm in relation to an obstacle of 100 cm in diameter and a door of 75 cm) was not sufficiently large to see significant variations in the analyzed crowd quantities.
